# Influence of spinal anesthesia versus general anesthesia on postoperative delirium in patients with hip fractures: A systematic review and meta-analysis

**DOI:** 10.1097/MD.0000000000044000

**Published:** 2025-08-29

**Authors:** Shuang Wu, Wan-Ping Ma, Hui Wang

**Affiliations:** a Operating Theater, Beijing Chaoyang Hospital, Capital Medical University, Beijing, China; b Anesthesiology Department, Beijing Chaoyang Hospital, Capital Medical University, Beijing, China.

**Keywords:** general anesthesia, hip fracture surgery, meta-analysis, postoperative delirium, spinal anesthesia, systematic review

## Abstract

**Background:**

Anesthesia type for hip fracture surgery and its relation to postoperative delirium (POD) is a topic of ongoing debate. This study aimed to elucidate whether there is a significant difference in POD incidence, mortality, and length of hospital stay between general anesthesia (GA) and spinal anesthesia (SA).

**Methods:**

We conducted a systematic review and meta-analysis of randomized controlled trials that compared the outcomes of GA and SA in hip fracture surgeries, adhering to Preferred Reporting Items for Systematic Reviews and Meta-Analyses guidelines. Data was extracted from 4 databases: PubMed, Embase, Web of Science, and the Cochrane Library. The main outcome of interest was the incidence of POD, with secondary outcomes including length of hospital stay and mortality rate.

**Results:**

The initial database search yielded 1012 studies, 9 of which met inclusion criteria and were included in the meta-analysis. There was no significant difference in the incidence of POD at postoperative day 4 (RR = 1.03, 95% CI: 0.85–1.25, *P* > .05) or day 7 (RR = 1.05, 95% CI: 0.89–1.25, *P* > .05) between GA and SA groups. The mortality rate at 3 months post-surgery (RR = 1.02, 95% CI: 0.69–1.50, *P* > .05) and total hospital stay duration (MD = −0.04, 95% CI: −0.17 to 0.08, *P* > .05) also did not significantly differ between the 2 groups.

**Conclusion:**

Although SA has certain advantages over GA, no significant difference was observed in the incidence of postoperative delirium, mortality, and hospital stay length between the 2 methods.

## 1. Introduction

Postoperative delirium (POD) is an acute neuropsychiatric syndrome that typically develops within the first week after surgery, presenting a significant medical challenge.^[1]^ It is characterized by cognitive dysfunction and decreased attention, leading to an inability to focus or shift attention. In addition to cognitive impairment, POD may involve changes in perception, physical function, consciousness, sleep-wake cycle, and psychomotor behavior. This condition has gained increasing attention due to its prevalence and severe impact on patients, particularly those undergoing hip fracture surgeries.^[2]^

The global burden of hip fractures is rising alarmingly. The incidence, which was 1.26 million in 1990, is projected to reach 4.5 million by 2050, signaling a looming healthcare crisis.^[3]^ Among hip fracture patients, about 20% are expected to face postoperative complications such as delirium, disability, and cardiovascular diseases. These complications significantly affect patients’ prognosis and quality of life, highlighting the importance of early prediction, prevention, and management.

POD is of particular concern in hip fracture patients, with some estimates suggesting that up to 50% may experience it.^[4]^ POD is linked to various negative outcomes, including increased mortality, prolonged hospital stays, and other postoperative complications.^[5–7]^ These statistics underscore the need for effective strategies to prevent POD.

The use of different anesthesia techniques, particularly general anesthesia (GA) and spinal anesthesia (SA), has been debated in relation to POD prevention. GA induces unconsciousness and analgesia but may affect the central nervous system in ways that influence the incidence of POD. In contrast, SA, which administers local anesthetics into the subarachnoid space, primarily affects the lower body while preserving consciousness. Understanding the differential effects of these anesthetic methods on the central nervous system and their potential impact on POD is crucial.

Based on the existing literature, our research hypothesis is that SA will lead to a lower incidence of POD compared to GA in patients undergoing hip fracture surgeries. This hypothesis is grounded in the premise that SA, by avoiding the central nervous system depression seen with GA, might reduce the risk factors associated with POD.

In recent years, there has been growing interest in comparing the effects of GA and SA on POD in hip fracture patients. Despite several studies, results have been inconsistent, necessitating a comprehensive, evidence-based review. This study aims to conduct a systematic review and meta-analysis to examine how GA and SA influence POD in hip fracture surgeries. Additionally, we seek to explore the mechanisms by which different anesthetic techniques might modify the risk of POD and influence postoperative outcomes. Our findings will provide a clearer understanding of the relationship between anesthesia type and POD, offering valuable insights for clinical decision-making and improving patient care in this vulnerable population.

## 2. Materials and methods

### 2.1. Search strategy

In conducting our systematic review and subsequent report of the findings, we diligently adhered to the recommendations outlined in the Preferred Reporting Items for Systematic Reviews and Meta-Analyses guidelines.^[8]^ We utilized 4 electronic databases for our data retrieval: PubMed, Embase, Web of Science, and the Cochrane Library. The search was conducted on January 6, 2024, and was not constrained by any specific timeline, thereby ensuring a comprehensive coverage of all relevant research. We tailored our vocabulary and syntax to suit the specific requirements of each database, ensuring optimal results. In PubMed, our search strategy incorporated the following terms: Hip Fractures, femur fracture, Delirium, Cognitive Dysfunction, Postoperative Cognitive Complications, randomized controlled trial. Our search strategy was not restricted by language, thereby expanding our scope to include all relevant research irrespective of the language of publication. In addition to the systematic electronic search, we also manually scrutinized the reference lists of the identified articles. This additional step was taken to uncover any potentially valuable studies that may have been missed in the initial database search. Our aim was to leave no stone unturned in our effort to compile a comprehensive body of evidence to underpin this meta-analysis.

### 2.2. Inclusion criteria

Studies included in the systematic review needed to meet the following criteria – Study design: Only randomized controlled trials (RCTs) will be considered for inclusion, irrespective of the language of publication; Population: The study subjects must be patients who have undergone surgery due to hip fractures, and they should be aged 18 years or older; Intervention: The studies included should involve the use of SA and GA as the intervention procedures; Outcome measures: The primary outcome to be evaluated is the incidence of POD. The secondary outcomes include total hospitalization time and mortality rate. If a study involving the same population is published more than once, select the study with the largest sample size or the newly published study.

The exclusion criteria were as follows: Literature that has been published on multiple occasions; studies that present incomplete, unclear, or inconsistent analytical data and outcome measures; studies of substandard quality or those missing original data; manuscripts including case reports, commentaries, expert opinions, and narrative reviews.

### 2.3. Data extraction

The process of literature screening and data extraction will be independently undertaken by 2 evaluators, with the results cross-verified. Should any disagreements arise during this process, they should be addressed through discussions between the evaluators involved. If necessary, a third evaluator may be consulted for their insight. The primary data to be extracted includes the following – Basic information: This encompasses the title of the study, author(s), country of origin, and year of publication; Characteristics of included studies: Details such as sample size, age and gender of participants, surgical and anesthetic techniques employed will be collected; Outcome measures: The specific tools used for the assessment of delirium will be noted. In the event that a published report does not contain the necessary data, we will reach out to the investigators of the original study via email to request the unreported data. This approach ensures comprehensive data collection and thereby contributes to the thoroughness and reliability of our meta-analysis.

### 2.4. Quality assessment

The integrity of the studies included in this meta-analysis was scrutinized utilizing the Cochrane Collaboration’s tool for assessing the risk of bias.^[9]^ Two reviewers independently evaluated the following domains: random sequence generation, allocation concealment, blinding of participants and personnel, incomplete outcome data, selective reporting, and other potential sources of bias. Each of these domains was classified as having a low, unclear, or high risk of bias. In the event of differing opinions between reviewers, resolution was sought through discussion, or if required, by consulting a third reviewer.

### 2.5. Statistical analyses

Inter-study heterogeneity was evaluated utilizing chi-square statistics, with the degree of heterogeneity quantified by the *I*^2^ value. An *I*^2^ value below 50%, coupled with a corresponding *P*-value equal to or above .10, indicated the absence of significant heterogeneity, warranting the use of a fixed-effect model to calculate the combined effect size. Conversely, when the *I*^2^ value was at least 50%, or the associated *P*-value was <.10, significant heterogeneity was inferred. Under such circumstances, a subgroup analysis or sensitivity analysis was performed to pinpoint and address potential sources of heterogeneity. If statistical heterogeneity alone was present, a random-effects model was adopted for the computation of the combined effect size. The potential for publication bias was evaluated by assessing the symmetry of the funnel plot and through Egger’s test. All statistical tests employed were 2-sided, and a *P*-value of <.05 was considered to indicate statistical significance. The statistical analyses were carried out using Stata version 17 (StataCorp, College Station, TX).

## 3. Results

### 3.1. Search results and study selection

**Figure 1.**
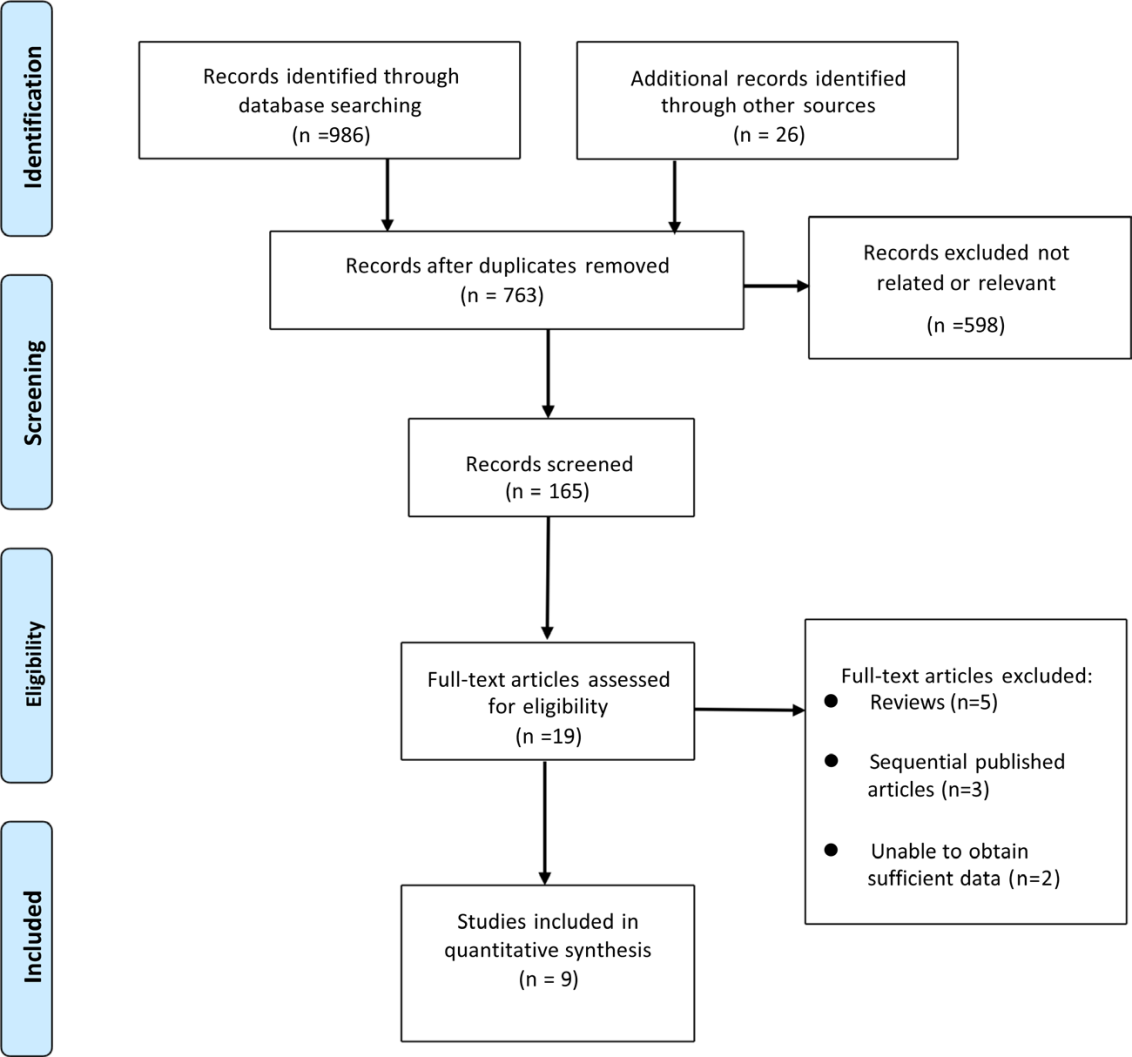
Selection process of included studies.

From the initial search of the electronic databases, 1012 related literatures were initially found. After removing repetitive literatures, reading titles and abstracts, and screening strictly according to the inclusion and exclusion criteria, 19 related literatures were obtained, and 10 were excluded from further reading. Finally, 9 articles were included.^[10–18]^ The literature screening process and results are shown in Figure 1.

### 3.2. Study characteristics

The characteristics of studies included in this systematic review are presented in Table 1. The included studies in this meta-analysis span multiple countries including Sweden, Japan, the United Kingdom, the United States, Greece, and China, reflecting a broad international scope. These studies, published between 1987 and 2022, encompass patient ages ranging from 50 to 105 years old. The studies compared 2 main types of anesthesia: SA, which includes spinal, epidural, or combined spinal epidural anesthesia, and GA. One of the Chinese studies in 2021 also incorporated lumbar sacral plexus nerve block (CLSB) alongside SA and GA. The sample sizes varied widely across these studies, with the smallest involving 12 participants and the largest encompassing 1600 participants. Each study maintained a balance in terms of the number of patients receiving SA and GA, allowing for a fair comparison. Furthermore, gender distribution within these studies varied, with both male and female subjects adequately represented. The primary observational index across all studies was the incidence of postoperative delirium. Additional outcomes such as total hospital stay and postoperative 3-month mortality were investigated in select studies. All the studies used standardized tools for the assessment of delirium, with the confusion assessment method (CAM) being the most commonly used, followed by the diagnostic and statistical manual of mental disorders, third edition (DSM-III) in one of the studies.

**Table 1:** Characteristics of studies included in the meta-analysis.

Included studies	Year	Country	Age (yr)	Sample size	Gender	Anesthesia method	Observational index	Delirium assessment tool
Li T^[10]^	2022	China	＞65	SA 471, GA 471	M 247, F 695	SA vs GA	Postoperative delirium, postoperative 3-month mortality	CAM
Neuman MD^[12]^	2021	USA	＞50	SA 795, GA 805	M 528, F 1072	SA vs GA	Postoperative delirium, total hospital stay, postoperative 3-month mortality	CAM
Tang LL^[11]^	2021	China	＞65	SA 55, GA 55	M 36, F 74	SA vs GA + CLSB	Postoperative delirium	CAM
Yang YP^[13]^	2019	China	66–90	SA 41, GA 41	M 32, F 50	SA vs GA	Postoperative delirium	CAM
Petros T^[14]^	2018	Greece	≥65	SA 37, GA 33	M 33, F 37	SA vs GA	Postoperative delirium, total hospital stay, postoperative 3-month mortality	CAM
Neuman MD^[15]^	2016	USA	＞57	SA 6, GA 6	M 9, F 3	SA vs GA	Postoperative delirium, postoperative 3-month mortality	CAM
Parker MJ^[16]^	2015	UK	52–105	SA 158, GA 164	M 87, F 235	SA vs GA	Postoperative delirium, total hospital stay, postoperative 3-month mortality	CAM
Kamitani K^[17]^	2003	Japan	≥70	SA 19, GA 21	M 4, F 36	SA vs GA	Postoperative delirium	CAM
Berggren D^[18]^	1987	Sweden	＞64	SA 28, GA 29	M 46, F 11	SA vs GA	Postoperative delirium	DSM-III

CAM = confusion assessment method, CLSB = lumbar sacral plexus nerve block, DSM-III = diagnostic and statistical manual of mental disorders, third edition, GA = general anesthesia, SA = spinal anesthesia (spinal, epidural, or combined spinal epidural anesthesia).

### 3.3. Results of quality assessment

**Figure 2.**
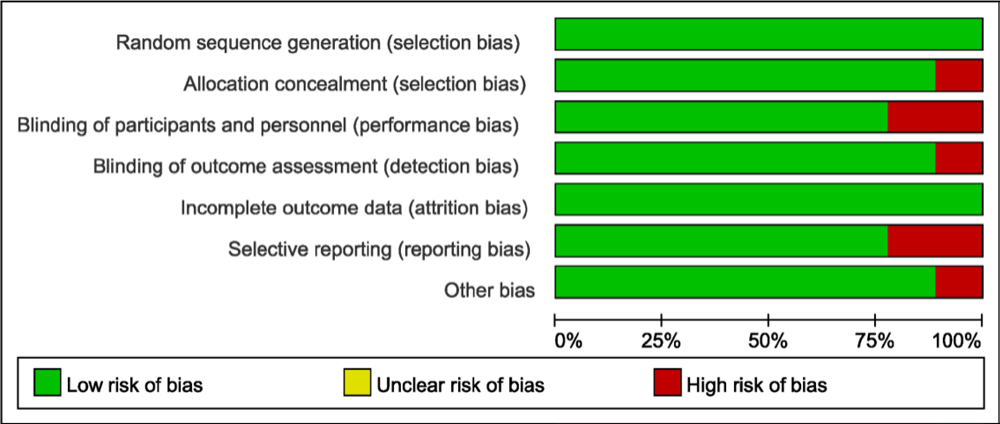
Quality assessment of included studies using cochrane collaboration’s tool criteria. Red in figure indicates high risk, yellow represents unclear risk and green means low risk.

A bias risk assessment was performed across various categories in the 9 included studies. High methodological integrity was observed in 3 studies, evidenced by a low risk of bias across all evaluated domains. Nonetheless, in 20% of the studies, a high risk of bias was identified in the realm of participant and personnel blinding, implying potential susceptibility to performance bias which could have impacted the study outcomes. Additionally, a considerable proportion of the included RCTs (21%) showed a high risk of bias due to selective reporting. This infers that the general results of these studies might have been influenced by incomplete or biased outcome reporting (Fig. 2).

### 3.4. Postoperative delirium rate at day 4

**Figure 3.**
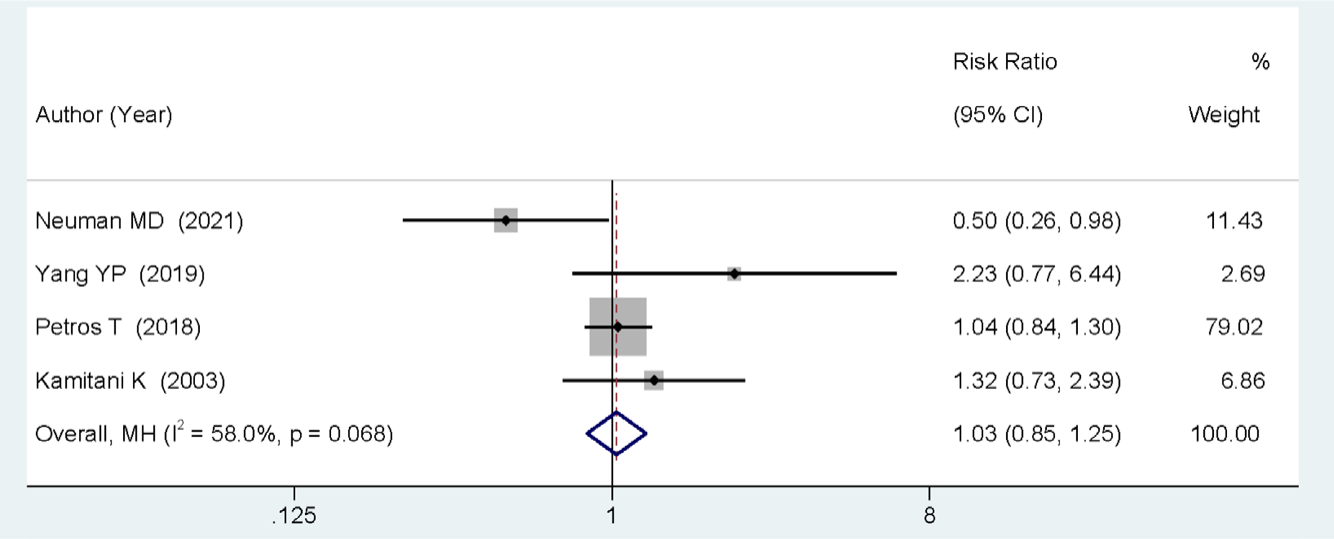
Forest plot comparing the incidence of delirium on postoperative day 4 between SA and GA. GA = general anesthesia, SA = spinal anesthesia.

Four studies reported on the occurrence rate of delirium 4 days after surgery using different anesthetic techniques. The heterogeneity of these 4 studies was significant (*P* = .068, *I*^2^ = 58.0%), thus a random-effects model was adopted for the meta-analysis. The analysis showed that there was no statistically significant difference between the 2 groups in terms of postoperative delirium occurrence at day 4 (RR = 1.03, 95% CI: 0.85–1.25, *P* > .05; Fig. 3).

### 3.5. Postoperative delirium rate at day 7

**Figure 4.**
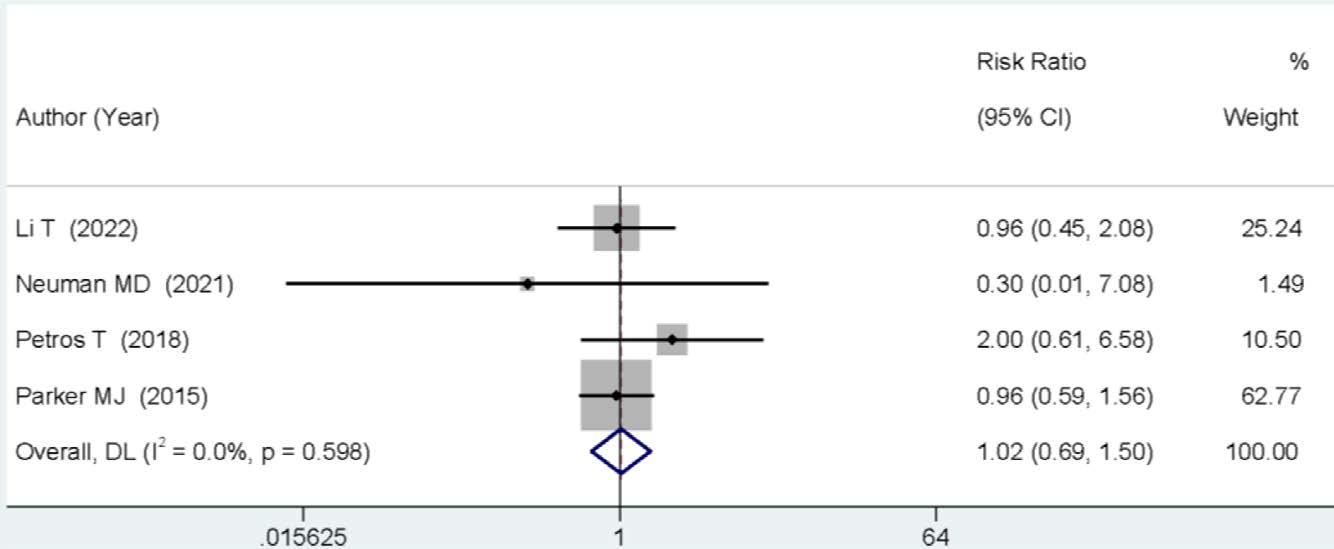
Forest plot comparing the incidence of delirium on postoperative day 7 between SA and GA. GA = general anesthesia, SA = spinal anesthesia.

Nine studies all reported on the delirium occurrence rate 7 days after surgery using different anesthetic techniques. The statistical heterogeneity of these 9 studies was relatively low (*P* = .298, *I*^2^ = 16.3%), and hence a fixed-effects model was utilized for the meta-analysis. The results showed no significant statistical difference in the occurrence rate of delirium on day 7 post-operation between the 2 groups (RR = 1.05, 95% CI: 0.89–1.25, *P* > .05; Fig. 4).

### 3.6. Postoperative 3-month mortality rate

**Figure 5.**
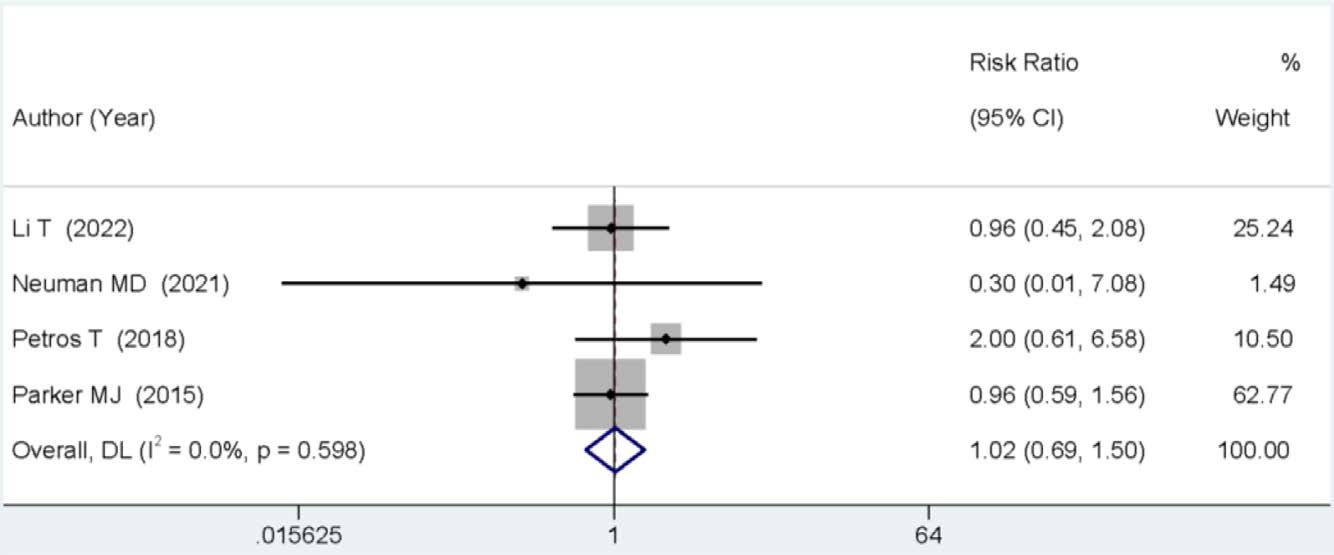
Forest plot comparing the mortality rate at 3 months post-surgery between SA and GA. GA = general anesthesia, SA = spinal anesthesia.

Four studies reported on the mortality rate occurring within 3 months after surgery. The meta-analysis showed no statistically significant difference in the mortality rate between the 2 groups (RR = 1.02, 95% CI: 0.69–1.50, *P* > .05; Fig. 5).

### 3.7. Total hospital stay duration

**Figure 6.**
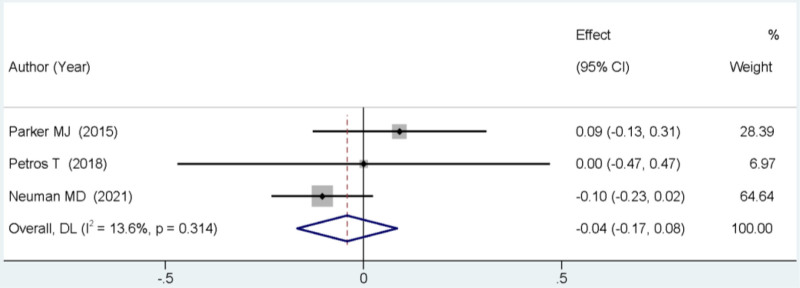
Forest plot comparing the total duration of hospital stay between SA and GA. GA = general anesthesia, SA = spinal anesthesia.

Three studies reported on the total duration of hospital stay. The meta-analysis revealed no statistically significant difference between the 2 groups in terms of the total length of hospital stay (MD = −0.04, 95% CI: −0.17 to 0.08, *P* > .05; Fig. 6).

### 3.8. Publication bias

**Figure 7.**
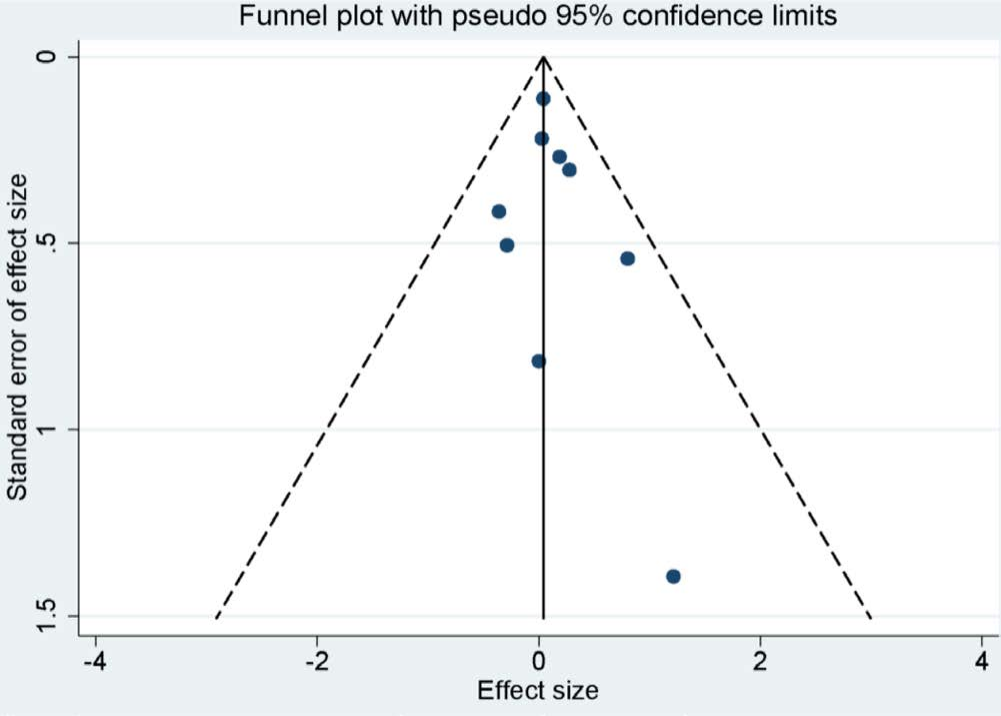
Funnel plot for publication bias in all included studies.

Constructed funnel plots from our included study demonstrated symmetry, indicating the absence of significant publication bias (Fig. 7). Further affirmation of this lack of notable bias was seen via the Egger’s linear regression test. This test yielded results indicating a nonsignificant publication bias in the conducted meta-analyses across varying variables (All *P*-values > .05). These findings serve to strengthen the integrity and robustness of the meta-analysis outcomes.

## 4. Discussion

The occurrence of postoperative delirium, especially in patients with hip fractures, has been a significant concern in clinical anesthesia practice. The cholinergic-dopaminergic imbalance theory posits that the principal mechanism underpinning delirium is the disturbed equilibrium in the cholinergic and dopaminergic systems.^[19]^ With aging, the brain function gradually deteriorates, leading to a decrease in acetylcholine transferase activity and a constant level of acetylcholinesterase activity. This imbalance eventually results in a reduction in the levels of the principal excitatory neurotransmitter, acetylcholine, and an increase in dopamine concentration, ultimately contributing to postoperative delirium. The risk factors for postoperative delirium, according to the 2017 European Anesthesia Guidelines, are primarily categorized into predisposing and triggering factors. Predisposing factors often include preexisting conditions or diseases such as advanced age, dementia, anemia, cardiovascular diseases, visual and auditory impairments, and alcohol abuse. Conversely, triggering factors typically include risk factors developed after a fracture such as infections, postoperative pain, sleep disturbances, hypoxemia, urinary retention, and constipation.^[20]^

Postoperative delirium has major clinical significance, particularly in elderly patients undergoing hip fracture surgery. The occurrence of delirium significantly affects patient recovery, leading to prolonged hospital stays and the potential for long-term cognitive dysfunction. This condition not only increases the risk of higher mortality but also contributes to a higher incidence of functional decline, thereby impairing the overall quality of life for these patients. In addition to the clinical burden on patients, postoperative delirium also increases healthcare costs due to extended hospital stays and the need for additional care. Given these challenges, preventing POD in hip fracture patients is crucial for improving patient outcomes, reducing healthcare expenditures, and enhancing the overall quality of life for elderly patients.

Currently, the primary anesthesia modalities for hip fracture patients are SA and GA. Past studies have suggested that the use of anticholinergic drugs and sedatives during GA may increase the risk of postoperative delirium. The imbalance in the brain’s neurotransmitter system induced by these anesthetic drugs may trigger postoperative delirium. Comparatively, SA offers some advantages such as reducing opioid usage, avoiding airway management, eliminating the need for endotracheal intubation, extending postoperative analgesia, and lowering the risk of postoperative nausea and vomiting.^[21,22]^

However, our meta-analysis did not find that SA significantly reduced the incidence of postoperative delirium in patients with hip fractures compared to GA, which is consistent with recent studies.^[10]^ This could be attributed to the multifactorial nature of postoperative delirium, including individual differences among the included patients, varied perioperative risk factors, underlying diseases, type of surgery, differences in anesthesia practices across countries, all of which could introduce some degree of bias in the results. The mortality rate after hip fracture surgery is primarily associated with high risk factors such as older age, severe underlying diseases, and in-hospital falls.^[23,24]^ In our meta-analysis, no significant difference was observed in the mortality rates within 3 months post-surgery between the 2 groups (*P* > .05). This could be due to differences in the age range of the included studies, the patients’ underlying diseases, and postoperative recovery. The analysis also showed no statistically significant difference in the total hospital stay between the 2 groups (*P* > .05), suggesting that factors such as the type of fracture, delay in surgery, in-hospital recovery, and economic conditions could influence the length of hospital stay.

It is important to note several limitations of our study. First, the primary outcome, postoperative delirium, was assessed using only the CAM, which may not fully capture the range of cognitive dysfunction in patients. The reliance on CAM and the subjective judgment of the assessor could introduce bias and limit the generalizability of the results. Second, some studies included in our analysis had smaller sample sizes, which may affect the robustness of the findings and leave room for positive results due to the limited statistical power. Third, in some studies, delirium was not the primary outcome, which may have resulted in incomplete or inconsistent data regarding its incidence and severity. Finally, while our study includes important RCTs, the quality of these studies varies, and high-quality, large-scale RCTs specifically examining the impact of different anesthesia techniques on postoperative delirium in hip fracture patients are still scarce. Only 9 studies met our inclusion criteria, and this limited number may influence the strength of the conclusions. Future research should aim to include larger, multi-center RCTs with better methodological quality to provide more definitive answers to the questions raised in this study.

## 5. Conclusions

While SA presents certain benefits over GA, our results suggest no significant difference in the incidence of postoperative delirium or other clinical outcomes such as mortality and length of hospital stay between the 2 methods. However, these findings should be interpreted in light of the multifactorial nature of postoperative delirium, with a variety of individual and procedural factors at play.

## Author contributions

**Conceptualization:** Shuang Wu, Hui Wang.

**Data curation:** Shuang Wu, Wan-Ping Ma.

**Formal analysis:** Shuang Wu, Wan-Ping Ma.

**Investigation:** Shuang Wu, Wan-Ping Ma.

**Methodology:** Shuang Wu, Wan-Ping Ma, Hui Wang.

**Resources:** Shuang Wu, Wan-Ping Ma.

**Software:** Wan-Ping Ma.

**Writing – original draft:** Shuang Wu.

**Writing – review & editing:** Hui Wang.
